# Mesh patch and anchors can improve clinical results of prosthetic replacement after resection of primary proximal humerus malignant tumor

**DOI:** 10.1038/s41598-020-78959-y

**Published:** 2021-01-12

**Authors:** Yongkun Yang, Yuan Li, Weifeng Liu, Xiaohui Niu

**Affiliations:** Department of Orthopedic Oncology Surgery, Beijing Ji Shui Tan Hospital, Peking University, Beijing, People’s Republic of China

**Keywords:** Cancer, Bone cancer, Cancer therapy, Sarcoma

## Abstract

The purpose of this study was to evaluate the functional results, complications and related factors of prosthesis reconstruction after malignant tumor resection of primary proximal humeral, and also evaluate whether soft tissue reconstruction with mesh patch and anchors can improve clinical results. From 2002 to 2016, forty-one patients were enrolled in this study. The pathological diagnosis contained 27 cases of osteosarcoma, 7 cases of chondrosarcoma and other primary malignant bone tumors. Both mesh patch and anchors were used in the reconstruction of joint capsule and the surrounding soft tissues in 27 cases. The mean postoperative follow-up was 60.6 months. The average active abduction angle and passive abduction angle was 33.5 (5–71) degrees and 72.4 (52–104) degrees. The prosthetic humeral head displacement was over 2 cm in 5 cases (12.2%). The average MSTS score was 23.1. The overall 5-year survival rate of prosthesis was 88.2%. The length of osteotomy, whether preserving deltoid muscle, whether applying mesh patch and anchors had significant effects on the abduction angle of shoulder joint; the length of osteotomy, whether applying mesh patch and anchors had significant effects on the degree of upward displacement of prosthesis. The application of both mesh patch and anchors in prosthesis reconstruction achieved more stable result and better function of shoulder joint. To ensure the stability of shoulder joint and the firm wrapping of surrounding soft tissue are key factors affecting the postoperative function.

## Introduction

The primary malignant bone tumor represented by osteosarcoma is mainly located in the skeleton of extremities, and the proximal humerus is the third most common site except distal femur and proximal tibia^1^. Shoulder joint and upper limb function are particularly important for patients' daily life. Therefore, it is an important topic to select appropriate limb saving method to reconstruct shoulder joint structure and function. For malignant bone tumors, the purpose of surgical treatment is to completely remove the tumor and achieve wide resection boundary. At the same time, we need to consider the reconstruction of bone and soft tissue defects after resection and also the restoration of shoulder joint function. The reconstruction methods after resection of proximal humeral tumor include endoprosthesis, allogeneic bone and joint transplantation^2–4^, composite of allogeneic bone and artificial prosthesis, autogenous bone transplantation^5–9^, arthrodesis^10^, tumor bone inactivation and replantation^11,12^.

At present, the most commonly used method is prosthetic replacement^13–15^. The failure rate of early prosthesis reconstruction was high. It took 8–10 weeks to design and manufacture customized prosthesis. It was difficult to accurately predict the actual length and thickness of the excised bone before operation. In the early 1980s, the appearance of module prosthesis changed the prosthesis reconstruction. The real length of bone defect was measured during the operation, and the most appropriate component was selected for reconstruction. Ross et al.^16^ reported 25 cases of proximal humeral prosthesis reconstruction with high rate of complications. The difficulty of operation mainly came from the matching of prosthesis and defect site, as well as tissue scar caused by preoperative biopsy and radiotherapy. Since then, cases have been reported successively, such as the studies reported by Kumar et al.^17^, Bickels et al.^18^ and Asavamongkolkul et al.^19^. The postoperative complications have been significantly reduced. The most complications were loosening and dislocation of the prosthesis. The postoperative function of shoulder joint is acceptable except the difficulty in lifting the shoulder.

Compared with the study of prosthesis reconstruction after resection of malignant bone tumors around the knee joint, there are less study reports of shoulder prosthesis reconstruction after resection of proximal humeral malignant tumors. How to restore the shoulder joint function and control the incidence of dislocation after prosthetic replacement is worthy of study. We improved the traditional prosthesis reconstruction by the application of both non-absorbable patch (more economical than artificial ligament) and anchors at the same time: the prosthesis was wrapped with mesh patch to restore the joint capsule and suturing the surrounding muscles on the patch; four anchors were fixed in different directions of the shoulder glenoid to increase the relative stability of prosthetic humeral head and shoulder glenoid. Therefore, the purpose of this study is to evaluate the functional results, complications and related factors of prosthesis reconstruction after resection of primary proximal humeral malignant tumor, and also evaluate whether soft tissue reconstruction with mesh patch and anchors can improve clinical results.

## Materials and methods

### General characteristics

All cases were from the musculoskeletal tumor database in our department. This study was approved by the Ethics Committee of Beijing Ji Shui Tan Hospital, and informed consent was obtained from all patients. All methods were carried out in accordance with relevant guidelines and regulations. The operation time was from 2002 to 2016. Criteria for inclusion were as follow: the tumor was located in the proximal humerus; pathological diagnosis was confirmed as primary malignant bone tumor; the initial operation was performed in our hospital; intra-articular tumor resection and prosthesis reconstruction was performed; there was completed clinical data; the postoperative follow-up was more than 24 months. According to the above criteria, 41 patients were enrolled in this study (Table 1). There were 19 males and 22 females with an average age of 29.2 (13–74) years. The pathological diagnosis contained 27 cases of osteosarcoma, 7 cases of chondrosarcoma, 2 cases of Ewing's sarcoma, 2 cases of undifferentiated pleomorphic sarcoma (UPS), 1 case of osteoleiomyosarcoma, and 2 cases of undifferentiated malignant tumor.

**Table 1:** Clinical characteristic of the patients.

No.	Gender	Age	Diagnosis	Length of humeral osteotomy (cm)	Preserving deltoid muscle	Mesh patch and anchors	Active abduction angle	Prosthesis failure/reason
1	Female	24	Osteosarcoma	16	Yes	No	26	No
2	Male	18	Osteosarcoma	31	No	No	20	Yes/dislocation
3	Male	39	Osteosarcoma	14	Yes	Yes	21	No
4	Female	17	Osteosarcoma	15	Yes	Yes	52	No
5	Male	18	Osteosarcoma	12	No	No	14	No
6	Female	39	Undifferentiated malignant tumor	12	No	No	32	Yes/loosening
7	Male	44	UPS	11	Yes	Yes	44	No
8	Female	53	UPS	18.5	Yes	No	32	No
9	Female	19	Osteosarcoma	15.5	Yes	No	35	No
10	Male	17	Osteosarcoma	18	Yes	No	32	No
11	Female	51	Chondrosarcoma	8	Yes	No	51	No
12	Male	46	Chondrosarcoma	19	Yes	No	34	No
13	Female	16	Osteosarcoma	12.5	Yes	No	28	No
14	Female	25	Osteosarcoma	20	Yes	Yes	25	No
15	Male	38	Chondrosarcoma	16	No	No	10	Yes/dislocation
16	Male	21	Osteosarcoma	16	No	No	16	No
17	Male	27	Undifferentiated malignant tumor	20	Yes	Yes	58	No
18	Female	38	Osteosarcoma	11.5	Yes	Yes	40	No
19	Male	16	Osteosarcoma	14	Yes	No	23	No
20	Female	34	Osteosarcoma	16	Yes	Yes	48	No
21	Female	39	Osteosarcoma	16	No	No	5	No
22	Female	19	Osteosarcoma	13	Yes	Yes	33	No
23	Female	29	Chondrosarcoma	14.5	Yes	Yes	40	Yes/recurrence
24	Male	16	Osteosarcoma	16	Yes	Yes	34	No
25	Female	13	Osteosarcoma	17	Yes	Yes	31	No
26	Male	18	Osteosarcoma	18	Yes	Yes	35	No
27	Female	16	Osteosarcoma	18	Yes	Yes	30	No
28	Female	22	Osteosarcoma	14	Yes	Yes	52	No
29	Female	42	Chondrosarcoma	10	Yes	Yes	33	No
30	Female	16	Osteosarcoma	15	Yes	Yes	42	No
31	Male	17	Ewing's sarcoma	32	Yes	Yes	20	No
32	Female	25	Osteosarcoma	17	No	Yes	25	No
33	Male	15	Osteosarcoma	18	Yes	Yes	71	No
34	Female	74	Osteoleiomyosarcoma	11	No	Yes	18	No
35	Male	49	Chondrosarcoma	13	Yes	Yes	36	No
36	Male	15	Osteosarcoma	23	No	Yes	20	No
37	Male	70	Chondrosarcoma	13.5	Yes	Yes	38	No
38	Male	17	Osteosarcoma	17.5	No	Yes	28	No
39	Female	41	Osteosarcoma	13	Yes	Yes	37	No
40	Female	26	Osteosarcoma	14	Yes	Yes	49	No
41	Male	17	Ewing's sarcoma	19	Yes	Yes	55	No

*UPS* undifferentiated pleomorphic sarcoma.

### Clinical diagnosis

**Figure 1:**
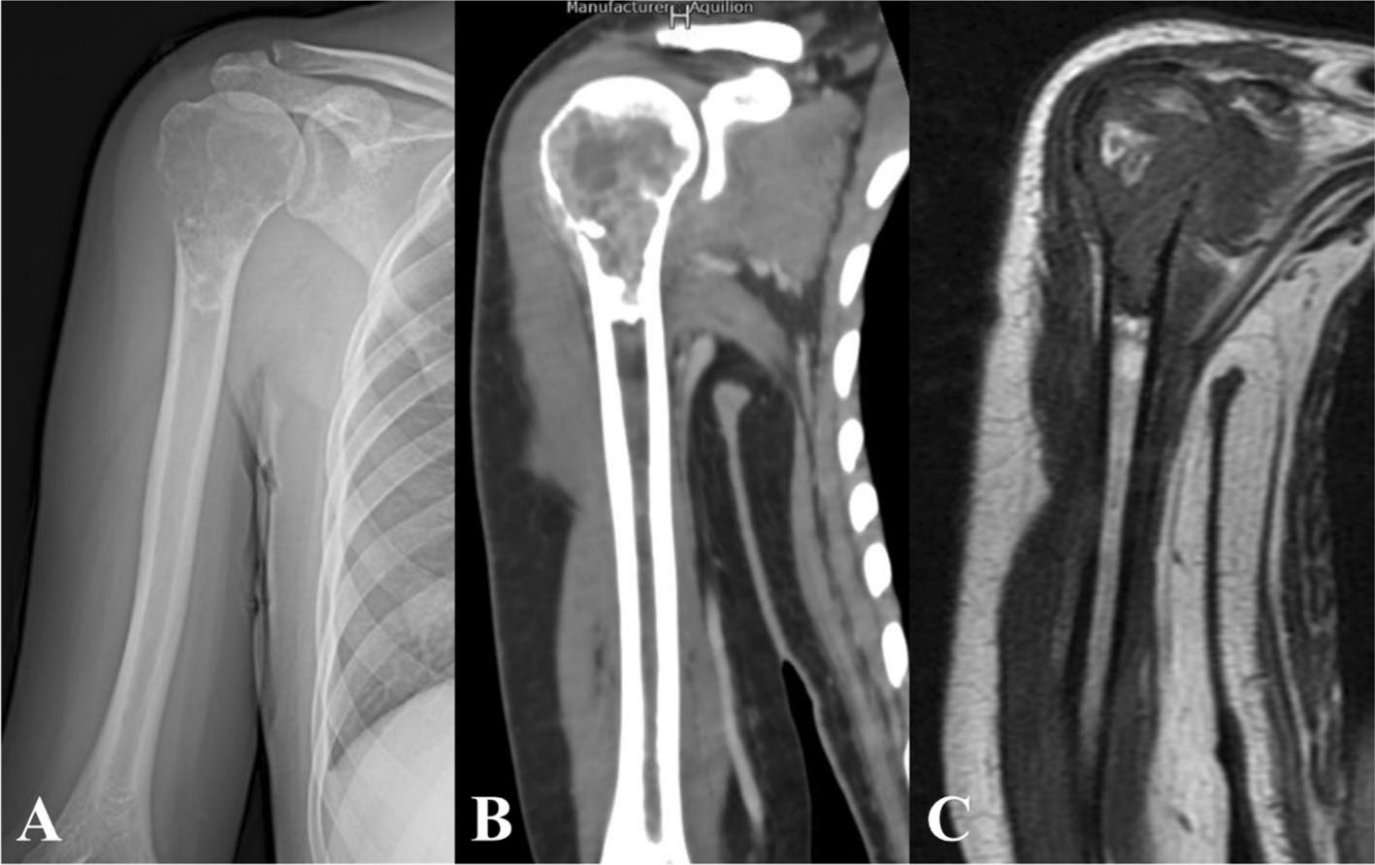
The preoperative humerus radiography (**A**), CT (**B**) and MRI (**C**) of a patient with proximal humerus osteosarcoma. The extent of tumor resection and length of osteotomy was determined according to preoperative imaging examinations.

All patients received radiography, CT and MRI of proximal humerus (Fig. 1) and whole body bone scanning before operation. The diagnosis was confirmed as primary malignant bone tumor by preoperative biopsy. The patients with osteosarcoma and Ewing sarcoma received preoperative chemotherapy.

### Surgical treatment

**Figure 2:**
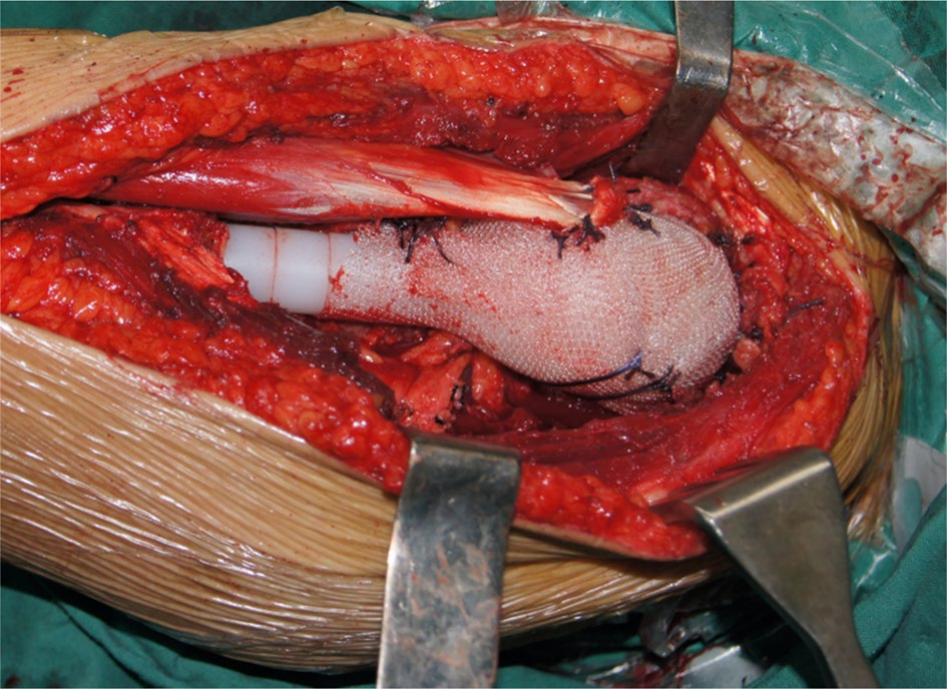
The intraoperative photograph showed that the prosthesis was wrapped with mesh patch to repair the joint capsule and then the surrounding muscles were sutured on mesh patch.

**Figure 3:**
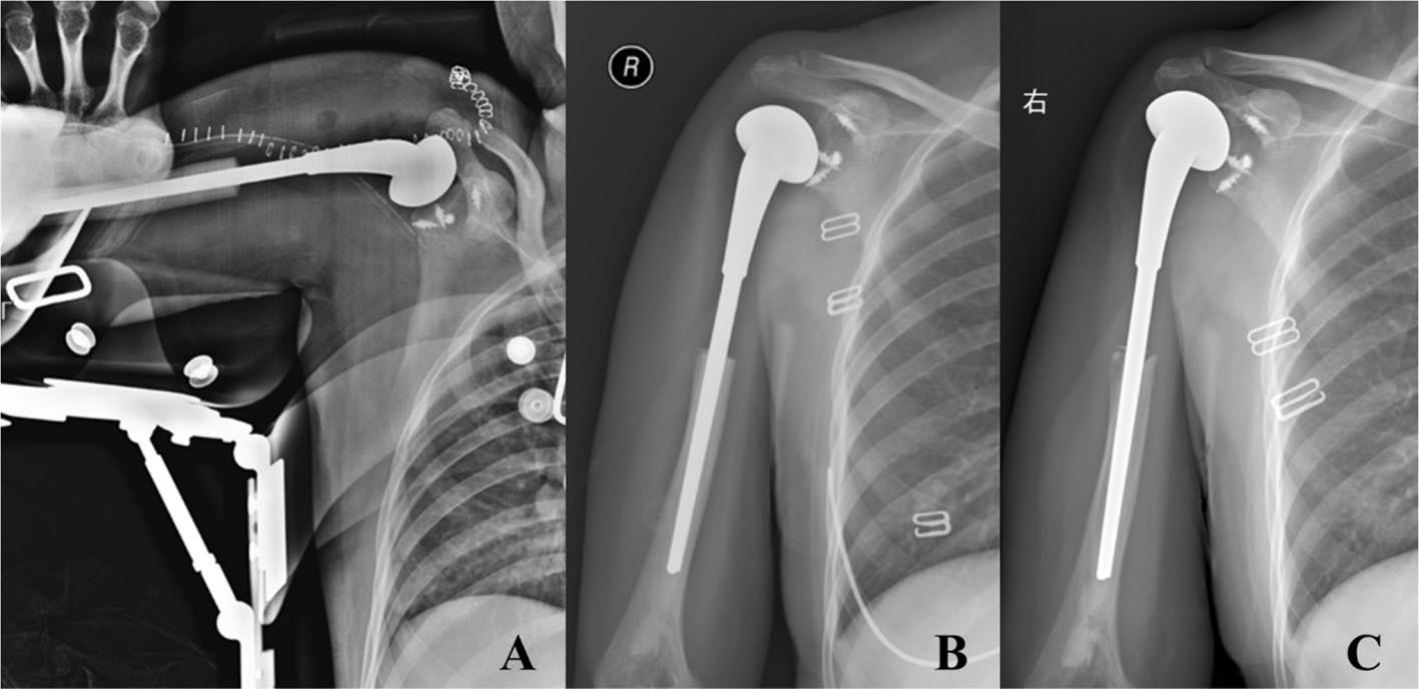
The postoperative radiography (**A**) showed 4 anchors were fixed in different directions (upper, lower, front and back) of the glenoid to restore the stability of the prosthesis. The radiography 3 months postoperative (**B**) and the radiography 104 months postoperative (**C**) showed the prosthesis was in stable position without obvious loosening and dislocation.

The patient was in supine position and the anterior incision of proximal upper arm was performed. The original biopsy pathway was resected. The resection range was determined according to the preoperative imaging examinations. The osteotomy plane of the humerus was design as 3–5 cm away from the edge of tumor and thus the extensive resection of the proximal humerus tumor was performed. After resection, the shoulder prosthesis (Chunli Zhengda Technology Co., Ltd) reconstruction was performed and the prosthesis was fixed by bone cement. The non-absorbable mesh patch and anchors were not used in the early cases. In the later stage, the traditional method of prosthesis reconstruction was innovated. The non-absorbable mesh patch and anchors were used in the reconstruction in 27 cases. The prosthesis was wrapped with mesh (Bard Crurasoft Patch) to repair the joint capsule, and the surrounding muscles such as pectoralis major, biceps brachii, brachii and triceps brachii were sutured on mesh patch (Fig. 2). On this basis, four anchors (DePuy Mitek FASTIN) were fixed in different directions (upper, lower, front and back) of the glenoid to improve the postoperative relative stability of the prosthetic humeral head and the shoulder glenoid (Fig. 3).

### Postoperative treatment and follow-up

The drainage was continued until the drainage volume was less than 50 ml/day. The suture was removed two weeks after operation. The shoulder joint was fixed in the abduction flexion position for 8–12 weeks. During which the muscle isometric contraction of and the exercise of elbow and wrist joints were performed. Postoperative chemotherapy was performed for osteosarcoma and Ewing sarcoma. The abduction bag was removed for exercise of shoulder joint function after 8–12 weeks postoperative.

The mean postoperative follow-up time was 60.6 months (median 57, range 24–149 months). The patients were followed every 3 months after operation in the first 2 years, 4 months between the third and fifth years, and 6 months after the fifth year. The reexamination items included radiography and CT of primary site and chest radiograph. Limb function was evaluated according to MSTS scoring system.

### Statistical analysis

SPSS 22.0 (SPSS Corporation, USA) was used to analyze the data. Mean value t-test was used for comparison of measurement data and chi square test was used for comparison of counting data. Kaplan–Meier method was used to calculate the survival rate of prostheses. Log rank test was used to compare the survival rate in different groups. Cox regression model was used for correlation analysis. P < 0.05 was defined as significant difference.

## Results

### General results

**Figure 4:**
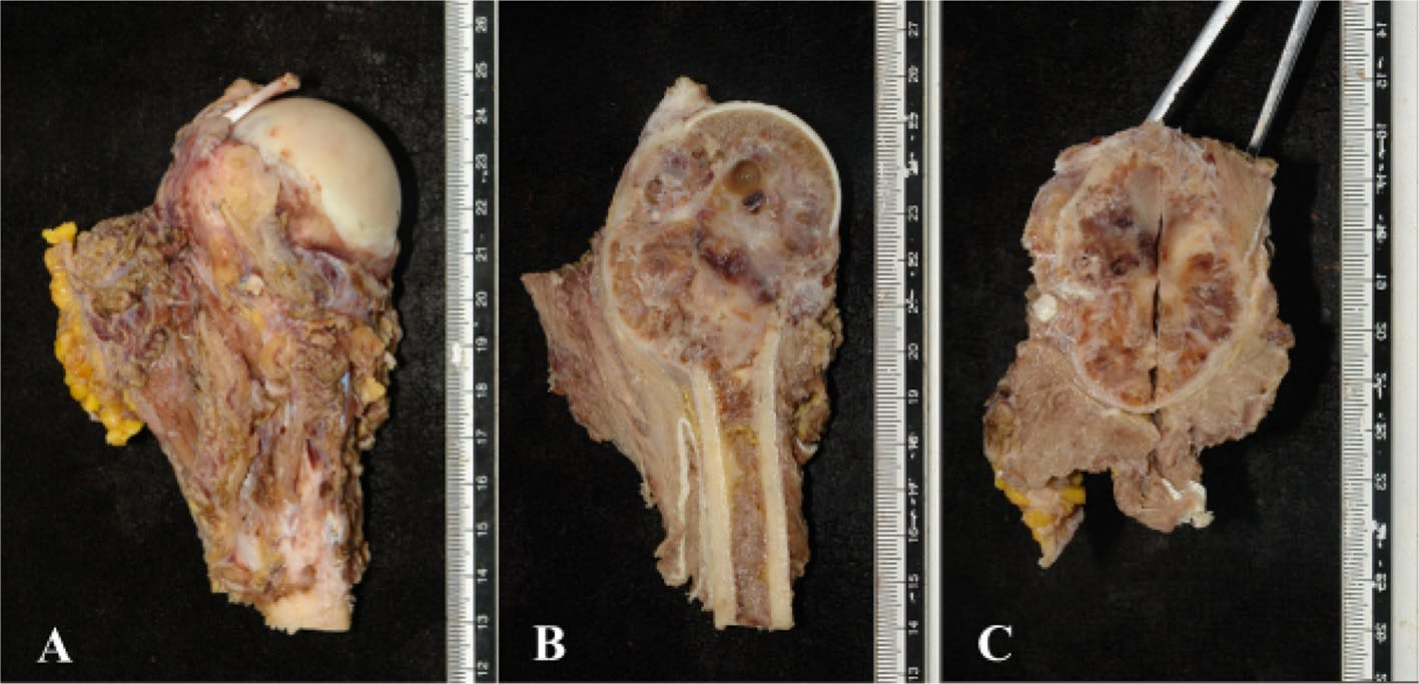
The postoperative specimen was soaked in formalin and then photographed (**A**). The specimen was cut along the longitudinal (**B**) and transverse (**C**) lines to analyze whether the safe surgical margin and osteotomy length were achieved.

The deltoid muscle was retained or partial retained in 31 cases. The average length of humeral osteotomy was 16.1 (8.0–32.0) cm (Fig. 4) and the average reconstruction length of prosthesis was 15.3 (7.0–31.0) cm. The average length of prosthetic stem was 10.0 (4.0–15.0) cm and the average diameter of prosthetic humeral head was 4.3 (3.6–5.0) cm.

### Shoulder joint mobility and function

**Figure 5:**
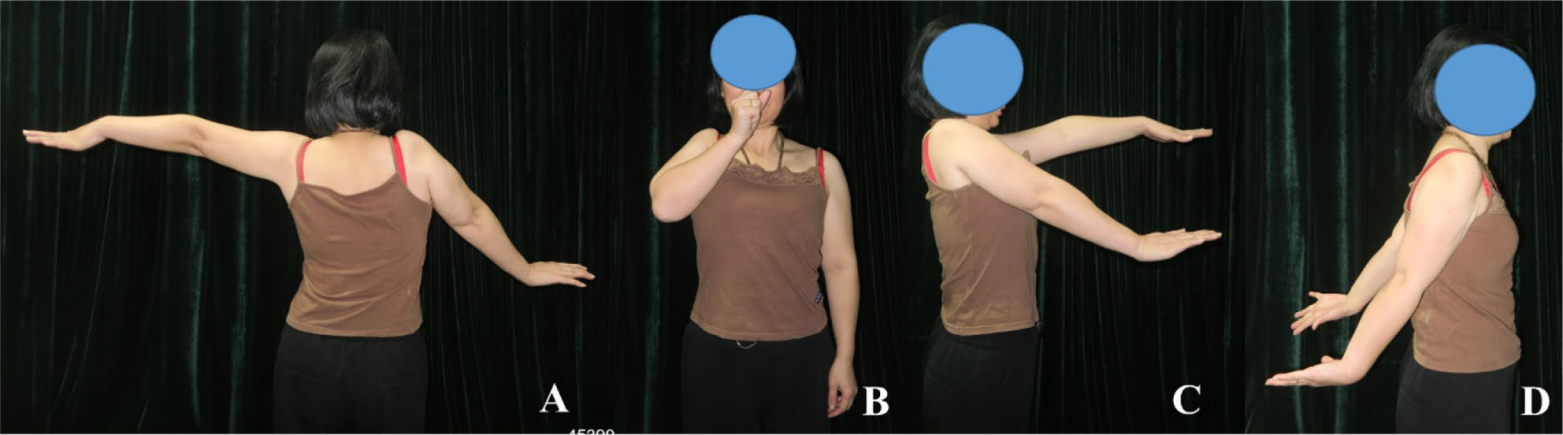
The photographs 10 years postoperative showed the function and the range of active movement of the shoulder joint.

Up to the latest follow-up, the average active abduction angle and passive abduction angle was 33.5 (5–71) degrees and 72.4 (52–104) degrees (Fig. 5). The average angle difference between active and passive abduction was 35.0 (0–65) degrees. The prosthetic humeral head moved upward with different levels in 34 cases with an average of 1.3 (0.4–4.1) cm. The displacement was over 2 cm in 5 cases (12.2%). The average MSTS score was 23.1 as follows: pain with 4.7, functional activities with 3.9, emotional acceptance with 4.0, positioning of the hand with 2.1, manual dexterity with 4.9 and lifting ability with 3.5.

### Prosthesis complications

Three cases received secondary operation due to prosthesis complications (Table 1). A 39 years old female received secondary operation due to prosthetic loosening 56 months after initial operation and the prosthesis was removed. Two cases underwent reoperation because of serious upward displacement of prosthesis: a 38 years old male underwent prosthesis revision 54 months after initial operation; another 18 years old male underwent prosthetic removal 82 months after initial operation. The mesh patch and anchors were not applied in the initial operation of these 2 cases.

**Figure 6:**
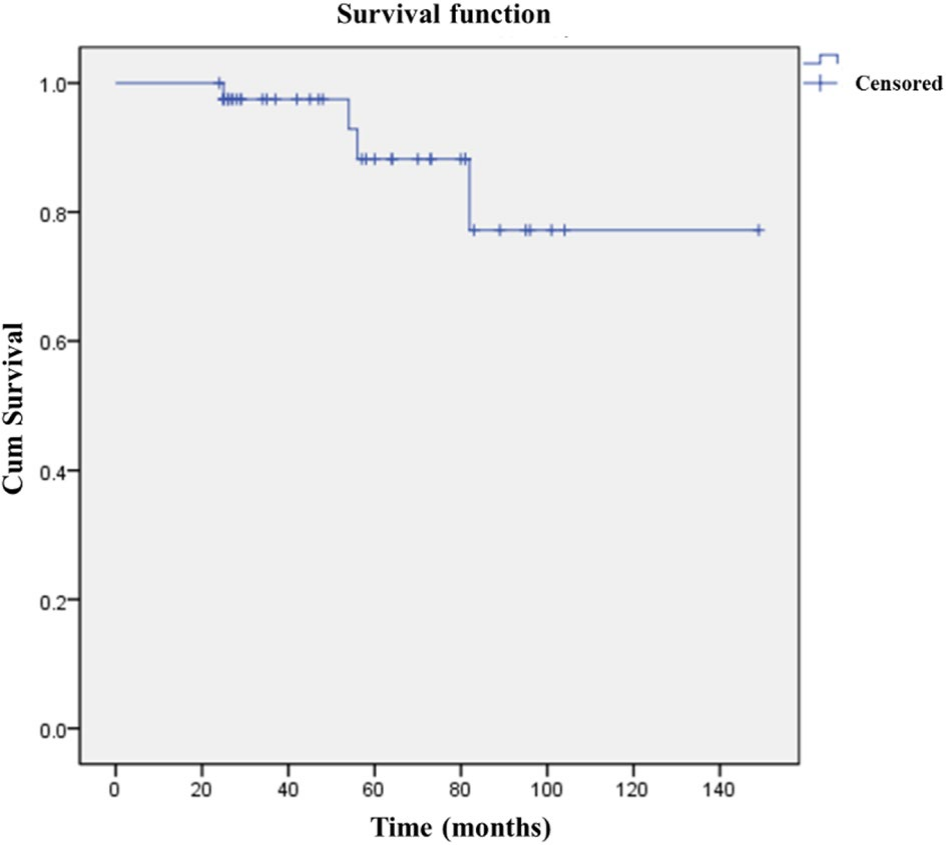
The survival curve of the prosthesis showed that the overall 5-year survival rate of prosthesis was 88.2% and the mean survival time of prosthesis was 129.8 (95% CI 111.9–147.7) months.

Amputation was performed in 1 case due to tumor recurrence. The overall 5-year survival rate of prosthesis was 88.2% and the mean survival time of prosthesis was 129.8 (95% CI 111.9–147.7) months (Fig. 6).

### The influencing factors of abduction angle of shoulder joint

The univariate analysis showed osteotomy length has significant influence on abduction angle (P = 0.047, t = 2.109). The average abduction angle in deltoid muscle preserved cases and not preserved cases were 38.2 (20–71) degrees and 18.8 (5–32) degrees (P = 0.000, f = 23.287). The average abduction angle in mesh patch and anchors applied cases and not applied cases were 37.6 (20–71) degrees and 25.6 (5–51) degrees (P = 0.005, f = 9.076).

The multivariate analysis showed that the length of osteotomy (P = 0.037, t = − 2.174), whether preserving deltoid muscle (P = 0.027, t = 1.825), whether applying mesh patch and anchors (P = 0.002, t = 3.444) had significant effects on the abduction angle of shoulder joint; the age (P = 0.847, t = 0.138), gender (P = 0.832, t = − 0.124) and size of humeral head (P = 0.633, t = − 0.483) had no significant effect on the abduction angle of shoulder joint.

### The influencing factors of upward displacement distance of prosthesis

The univariate analysis showed osteotomy length had significant influence on upward displacement (P = 0.006, t = 2.925). The average upward displacement distance in deltoid muscle preserved cases and not preserved cases were 0.97 (0–2.3) cm and 1.84 (0.4–4.1) cm (P = 0.011, f = 7.103). The average upward displacement distance in mesh patch and anchors applied cases and not applied cases were 0.93 (0–2.6) cm and 1.68 (0.4–4.1) cm (P = 0.014, f = 6.821).

The multivariate analysis showed that the length of osteotomy (P = 0.006, t = − 2.922), whether applying mesh patch and anchors (P = 0.005, t = 3.041) had significant effects on the degree of upward displacement of prosthesis; the age (P = 0.890, t = − 0.140), gender (P = 0.058, t = − 1.983), humeral head size (P = 0.578, t = 0.562), whether preserving deltoid muscle (P = 0.958, t = 0.053) and active abduction angle of shoulder (P = 0.108, t = − 1.656) had no significant effect on the degree of upward displacement of prosthesis.

### Oncology results

The local recurrences were found in 3 cases which included 1 case of osteosarcoma (3.7%) and 2 cases of chondrosarcoma (28.5%). The distant metastases were found in 8 cases. There were 6 cases of osteosarcoma (22.2%) which included 3 cases of lung metastasis, 1 case of bone metastasis, 1 case of lung and bone metastasis, 1 case of lung metastasis and kidney metastasis. The other 2 metastasis cases were 1 case of pelvis metastasis of osteoleiomyosarcoma and 1 case of bone metastasis of UPS.

## Discussion

Prosthetic replacement is the most widely used reconstruction method in the treatment of primary malignant bone tumor^13–19^. If enough muscles and soft tissues were preserved after tumor resection, the shoulder prosthesis can achieve good function. On the contrary, if there were not enough soft tissues, the prosthesis could only become the filler with poor function^20^, and the abduction angle of shoulder rarely reached 90°^21^. With the extensive application of prosthesis reconstruction and the improvement of surgical technology, the overall function and patient satisfaction are acceptable. But there are common problem of limited mobility, especially limited shoulder abduction^22,23^. In addition, postoperative joint instability and dislocation of the prosthetic humeral head are also common complications. The dislocation of prosthesis is often accompanied by obvious limitation of shoulder abduction, thus it is important to reconstruct the stability of shoulder with soft tissues. As the innovation of reconstruction method different from traditional operation, we used both mesh patch and anchors in the reconstruction of joint capsule and the surrounding tissues in some patients in the present study.

Our study showed the average angle of active and passive abduction of shoulder joint was 33.5 (5–71) degrees and 72.4 (52–104) degrees. The multivariate analysis showed that the length of osteotomy, whether preserving deltoid muscle, whether applying mesh patch and anchor had significant effects on the abduction angle of shoulder joint. Among the above significant factors, the length of osteotomy and whether to retain deltoid muscle are determined by the objective situation of tumor. The safe surgical margin cannot be changed. We should not deliberately retain the muscle or bone structure that should be removed together with the tumor; otherwise the local recurrence rate will increase. The application of mesh patch and anchors can be achieved by improving the operation method. We did not use mesh patch and anchors in early cases, but we used both mesh patch and anchors in the proximal humeral prosthesis replacement surgery in later cases and gradually achieved better results.

The stability reconstruction of shoulder joint includes not only the reconstruction of shoulder capsule related structures, but also muscle attachment around the proximal humerus and the balance of tension. Relevant improvement methods were proposed in previous reports. Wittig et al.^24^ used dynamic and static system to strengthen the prosthesis in 23 cases of proximal humerus osteosarcoma. The static system of cemented prosthesis was suspended from scapula and clavicle by 3 mm non-absorbable suture, the pectoralis major muscle was used as dynamic suspension system at the same time. All shoulder joints are stable and painless after operation. The complications included 8 cases of temporary paralysis and 1 case of peri-prosthetic fracture. Although the survival rate of prosthesis was high, but the mobility of shoulder joint was obviously limited. Early report by Ross et al.^16^ studied 24 cases and none of them received soft tissue reconstructed on the proximal humeral prosthesis. The degree of active flexion, extension and abduction of shoulder joint was less than 30°, so the postoperative function was poor without shoulder capsule and soft tissue repairing. There have been some reports^25–27^ which using synthetic mesh to reconstruct the joint capsule and rotator cuff tendon on the prosthesis, but the postoperative mobility of the shoulder joint is still poor. The active abduction angle was not significantly improved and most of them were less than 60°.

The present study showed that the prosthetic humeral head moved upward with different levels in 34 cases with an average of 1.3 cm and the severe displacement was only in 5 cases (12.2%). Two cases underwent reoperation because of serious upward displacement of prosthesis. The mesh patch and anchors were not applied in the initial operation of these 2 patients. The multivariate analysis showed that the length of osteotomy and whether applying mesh patch and anchors had significant effects on the severity of upward displacement of prosthesis. The above results indicate the value of applying mesh patch and anchors once again. It can not only improve the mobility of shoulder joint, but also can improve the postoperative stability of the shoulder joint and reduce the upward dislocation of the humeral prosthesis. The previous reports^14,21,28,29^ showed the incidence of shoulder instability and dislocation in patients who did not receive artificial ligament reconstruction after proximal humerus prosthetic replacement was about 20–50%. Therefore, some studies evaluated whether the application of artificial ligament or mesh can improve the stability of shoulder joint, but the results were not very satisfactory and discrepancy in different studies. Kumar et al.^17^ applied Mersilene TM ligaments in the prosthesis reconstruction of shoulder joint, but most patients still had different degrees of subluxation. Van de Sande et al.^25^ and Raiss et al.^26^ also reported the results of artificial ligament reconstruction around the proximal end of prosthesis. The incidences of subluxation were still over 40%, but few patients need revision operation due to serious dislocation.

The soft tissue reconstruction method in our study is different from that in previous reports, because we used anchors and mesh patch at the same time. The mesh patch is more economical than artificial ligament and can reduce the cost of surgical treatment. Usually we use four anchors in the reconstruction of shoulder joint, which are fixed in four directions (upper, lower, front and back) of the shoulder glenoid. This innovation can reconstruct the stability of joint capsule and increase the wrapping and strengthening effect on the proximal humeral prosthesis. The average MSTS score was 23.1 (77%) in our follow-up as follows. Although the function results were satisfactory, the score of lifting ability was still the lowest one in all the scoring. The previous reports^17,21–30^ showed the functional scores after proximal humerus prosthesis reconstruction ranged from 60 to 80%.

Our study showed the 5-year survival rate of prosthesis was 88.2% and the mean survival time of prosthesis was 129.8 (95% CI 111.9–147.7) months. The survival rate and time of prosthesis were satisfactory. There were four patients with prosthesis failures and three of them were mechanical failures, which including severe dislocation and loosening. The most common cause (50%) was severe dislocation of the prosthesis and they were early cases without the application of mesh patch and anchors. The previous reports (systematic review with 341 patients)^30^ showed large discrepancy of prosthesis survival in different studies which ranged from 38 to 100%. The incidence of complications was between 5 and 22% in different reports^17,21–30^.

There were 3 cases with local recurrence which included 1 case of osteosarcoma (3.7%) and 2 cases of chondrosarcoma (28.5%). The distant metastases were found in 8 cases: 6 cases of osteosarcoma (22.2%), 1 case of osteoleiomyosarcoma and 1 case of UPS. The overall recurrence rate is relatively low, especially for osteosarcoma. It is mainly related to the preoperative planning and safe surgical margin of tumor resection.

There are some limitations in this study. As a retrospective clinical study, patients were not randomly assigned to receiving mesh patch and anchors reconstruction or not. The time spanning of the study was long which is due to the low incidence rate of primary bone tumors such as osteosarcoma and Ewing's sarcoma. Although the overall function was satisfactory, there was always a certain mismatch between expectation and reality in the active abduction of shoulder joint. The limitation of abduction and shoulder lifting is still the functional short plate in majority patients with prostheses reconstruction after resection of proximal humeral tumors. But it is difficult to improve significantly under the premise of current surgical treatment and technology.

In conclusion, the present study provides relative large number cases of prostheses reconstruction after primary proximal humerus malignant bone tumors resection in a single institute, and analyzes the long-term results. Different from traditional operation, the application of non-absorbable both mesh patch (more economical than artificial ligament) and anchors in prosthesis reconstruction achieved better stability and function of shoulder joint. On the premise of ensuring safe surgical margin, to ensure the stability of the shoulder joint and the firm wrapping of the surrounding soft tissue are very important for the postoperative function recovery.
